# Hyperabundance of a subordinate herbivorous gastropod causes localised overgrazing within kelp beds

**DOI:** 10.1007/s00442-026-05970-x

**Published:** 2026-09-06

**Authors:** Scott D. Ling, Matthew Rose, Paula A. Ruiz-Ruiz, Sterling B. Tebbett

**Affiliations:** Institute for Marine and Antarctic Studies, University of Tasmania, Hobart, TAS 7004 Australia

**Keywords:** Herbivory, Periwinkle, *Lunella undulata*, Subtidal reef, Caging experiment

## Abstract

Herbivory is a fundamental biological process that can determine the structure and functioning of ecosystems. On temperate reefs worldwide, herbivory by grazing macro-invertebrates can cause extensive overgrazing of kelp beds when grazers reach hyperabundance. Rising to local dominance, typically it is the grazing activity of a single species amongst others that is responsible for overgrazed ‘barrens’. However, the potential for subordinate grazing species to impact kelp beds is less understood and often overlooked. Here we observe apparent ‘barren’ patches within kelp beds associated with local hyperabundance of a subordinate grazer, the turbinid periwinkle *Lunella undulata*, in eastern Tasmania. We demonstrate capacity for this periwinkle to overgraze by deploying a caging experiment on a ‘peri-barren’ patch, involving inclusion and exclusion treatments, plus cage and background control plots. Fleshy macroalgae proliferated in cages excluding periwinkles, reaching mean percentage cover of 47 ± 8% SE by end of the 117-day experiment, which was 5-fold, 6-fold, and 10-fold higher than in partially caged plots, open plots, and periwinkle inclusion cages, respectively. Indicating cascading effects of harvesting, legal-sized periwinkles were commercially harvested from the peri-barren during the study, leading to algal recovery across all treatments except for periwinkle inclusion cages where overgrazing persisted. Kelp bed recovery ensued as periwinkle biomass declined, while cover of fleshy algae increased over ~ 16 months. Reflecting the ongoing trophic and climate cascades in eastern Tasmania, we hypothesise that increased harvesting of the now dominant grazer, the range-extending urchin *Centrostephanus rodgersii*, has enabled localised competitive release of the subordinate periwinkle grazer.

**Supplementary Information:**

The online version contains supplementary material available at https://doi.org/10.1007/s00442-026-05970-x.

## Introduction

Globally, herbivory is a fundamental biological process that can strongly influence community dynamics (e.g., Hairston et al. 1960; Gruner et al. 2008). Understanding where, when and which herbivores can exert top-down control of vegetation, and ultimately shape landscapes, is of profound interest to ecologists. Dramatic examples include intensive grazing by herds of megafauna (e.g., wildebeest; Hopcraft et al. 2015) or plagues of invertebrates (e.g., locusts; Le Gall et al. 2019), whereby singular herbivorous species can rise to overwhelming dominance and cause overgrazing of primary producers due to extreme and disproportionate herbivore biomass. However, effects of subordinate herbivores and/or dominant herbivores when occurring at lower abundances can be less obvious but can cause persistent hysteresis effects despite reductions in herbivore biomass below the point at which overgrazing initially occurred (May 1977; Ling et al. 2015). As such, disentangling the influence of herbivore(s) can be ecologically complex, a challenge made greater by shifts in herbivore behaviour and population dynamics in the presence or absence of higher predators (including humans) on these systems (e.g., Fortin et al. 2005).

On temperate reefs worldwide, herbivory by benthic grazing macro-invertebrates can influence community dynamics, with sea urchins and gastropod molluscs having the greatest impacts on subtidal macroalgae (reviewed by Poore et al 2012). Most dramatically, overabundant sea urchin populations can cause extensive overgrazing of kelp beds and form an alternative stable state of urchin barren grounds over vast areas of reef (e.g., Filbee-Dexter and Scheibling 2014; Ling et al. 2015). Akin to monospecific herds, typically it is a single overgrazing urchin species that rises to local or regional dominance to form and maintain barrens (Ling 2008; Valentine and Edgar 2010; Ling et al. 2015). Such is the local to regional dominance of overgrazing by particular urchin species (e.g., Rose et al. 2026), that barrens are locally prefixed by the dominant species on the barren, e.g., on Australasian temperate reefs, the term ‘*Centro*-barrens’ is used to colloquially refer to barrens formed by *Centrostephanus rodgersii*; ‘*Helio*-barrens’ for those formed by *Heliocidaris erythrogramma* (or *H. tuberculata*), and ‘kina barrens’, a Māori term used to refer to overgrazed areas formed by the urchin *Evechinus chloriticus* (Miller et al. 2026). However, a multitude of subordinate grazing urchin and mollusc species are also typically present on these barrens or within kelp beds (Strain and Johnson 2009; Ling et al. 2018), with the capacity of these herbivores to influence local community dynamics often assumed unimportant.

Pioneering experiments examining subtidal herbivory by temperate urchins and molluscs were conducted by Fletcher (1987) on eastern Australian reefs near Sydney, who demonstrated that both urchins and limpets were capable of influencing reef community dynamics. Strongest effects were observed for urchins followed by more subtle effects of limpets (Fletcher 1987). Notably, nil effects were reported for Turbinid gastropods known as periwinkles, specifically *Australium tentiforme* and *Lunella torquata* (formerly *Turbo torquata*) (Fletcher 1987). However, due to experimental limitations as well as subsequent personal observations, this result for Turbinid gastropods was deemed to be inconclusive and it was suggested that further experimentation was required to evaluate if Turbinid gastropods modify algal communities (Fletcher 1987). Moreover, experimental densities and biomass of Turbinid gastropods were unclear in this previous experiment (Fletcher 1987), which is an important consideration given that the capacity of a species to markedly influence algal communities is invariably dependent on their local density and/or biomass (Hill et al. 2003; Wright et al. 2005; Ling et al. 2015). Thus, the capacity of gastropod molluscs to influence subtidal reef community dynamics, particularly when occurring at high local densities, has remained an open question.

Across temperate Australia, the most common subtidal species of periwinkle encountered is *Lunella undulata*, which, like other periwinkles, has been reported as having little or no influence on standing macroalgal communities including being frequently found on blades of the kelp with seemingly little obvious impact for its kelp host (Clarkson and Shepherd 1985). Reaching ~ 70 mm in shell length, *L. undulata* has an expansive vertical distribution, spanning the intertidal to a depth of ~ 18 m (Keane et al. 2014; Edgar et al. 2020), and is a generalist herbivore that consumes a wide range of fleshy seaweed and encrusting forms (Clarkson and Shepherd 1985; Smoothey 2008; Wernberg et al. 2008). Despite its seemingly benign mode of herbivory, notable aggregations of *L. undulata* up to 100 s of individuals m^−2^ (biomass exceeding several kg m^−2^) have been observed in association with open bare patches of reef, or along gullies and sand edges with minimum macroalgae present (Keough et al. 1993; Vanderklift and Kendrick 2004; Currie and Sorokin 2009; Shepherd and Edgar 2013; Keane et al. 2014). Further implicating *L. undulata* as an impactful grazer, it possesses unusually large central teeth in its radula, enabling it to rasp relatively deeply into tough brown algae or corallines (Worthington and Fairweather 1989). In aquarium conditions, *L. undulata* has been observed to consume a wide variety of habitat-forming brown algae and grow effectively on these diets for more than 9 months (Steinberg and van Altena 1992). Taken together, these observations suggest potential for the periwinkle *L. undulata* to influence subtidal reef community dynamics despite inconclusive initial experimental findings (Fletcher 1987).

Here we observe the emergence of ostensible ‘barren’ patches associated with local hyperabundance of the periwinkle *L. undulata* aggregating at a local density exceeding > 100 s per m^2^. These apparent grazed features of 10 s to 100 s m^2^ in size are hereafter termed ‘peri-barrens’ based on the high local density of *L. undulata* in the patches, but causation of these bare patches by periwinkles remained to be critically tested. By deploying a caging experiment consisting of inclusion and exclusion cage treatments, plus cage and open control plots across a large peri-barren, we test the hypothesis that the typically subordinate herbivore *L. undulata* can exert a dominant influence on the reef community when occurring at high local densities.

## Materials and methods

### Study site

**Fig. 1:**
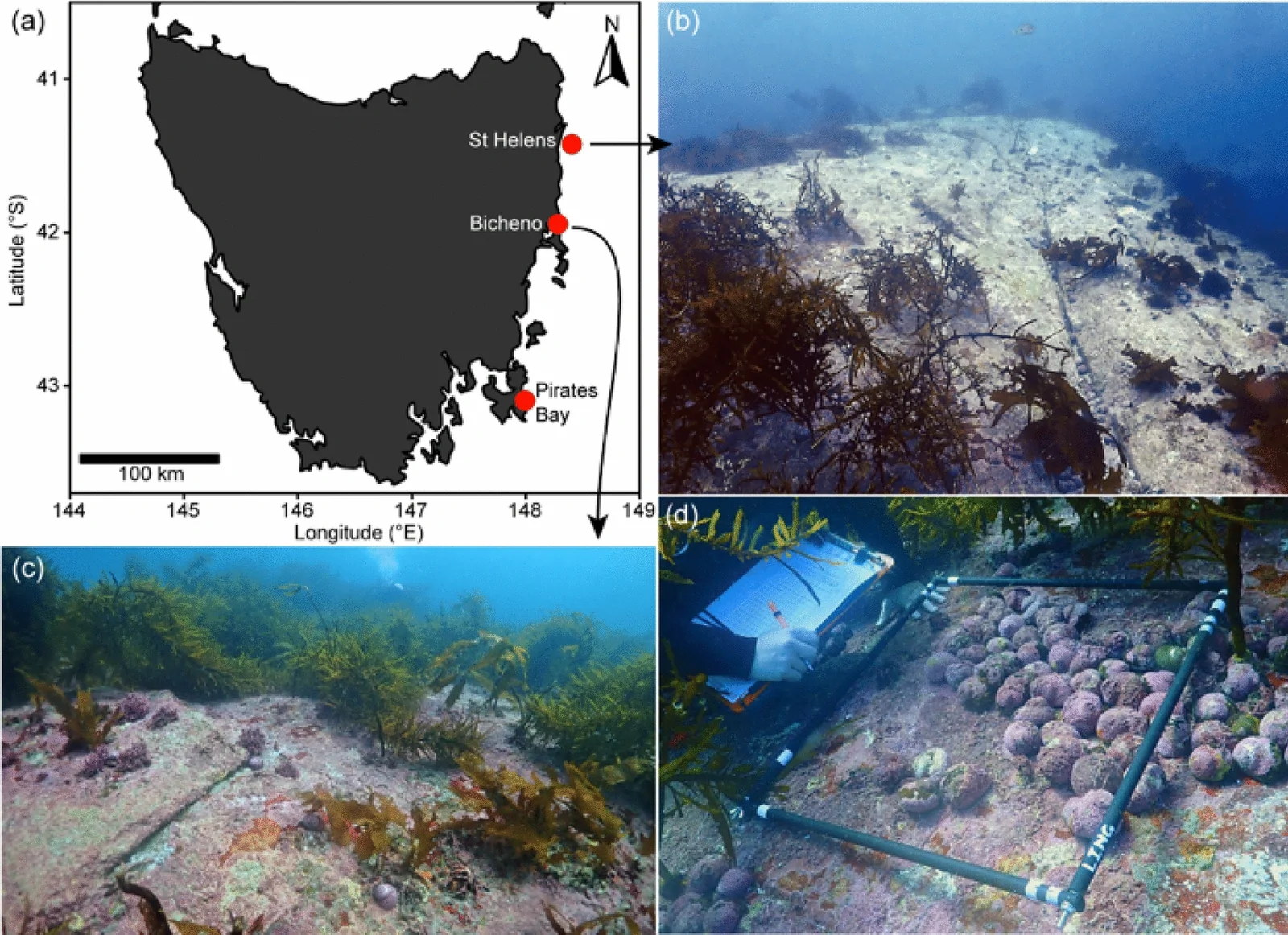
Observations of *Lunella undulata* hyperabundance and apparent ‘peri-barrens’. **a** Map indicating sites in eastern Tasmania where localised peri-barrens have been observed within mixed *Phyllospora comosa* and *Ecklonia radiata* kelp beds, **b** panorama of a peri-barren (~ 12 × 4 m) at St. Helens Island in July 2023 at 10-m depth, where the caging experiment was located (S41.3437; E148.3401, see Fig. S1 for detailed map), **c** panorama of a peri-barren (~ 3 × 2 m) at Bicheno in May 2022 at 9-m depth, **d** hyperabundance of large *L. undulata* (> 55 mm shell-length) at the edge of the peri-barrens at St. Helens Island, quadrat is 0.5 × 0.5 m in size containing an estimated periwinkle biomass of 1680 g (~ 6,700 g m^−2^) at 10-m depth (photographs SD Ling)

Initial emergence of apparent *L. undulata* ‘peri-barrens’ was observed at St. Helens Island in northeastern Tasmanian on 26 March 2023 (Fig. 1a, b; see Fig. S1 for detailed map). The most striking feature of the St. Helens Island study site is the extensive urchin barrens ranging from ~ 12 to 30 m depth as formed by the range-extending *C. rodgersii* in recent decades (Ling & Keane 2024). This specific site on the northwestern side of the island had been monitored for sea urchin abundance since 2001 and has been the site of ongoing experimental urchin research since 2003 (e.g., Ling et al. 2008; Ling and Johnson 2009; Rose et al. 2026). While the abundances of overgrazing urchins (*Centrostephanus rodgersii* and *Heliocidaris erythrogramma*) plus other large benthic macroinvertebrates, including abalone (*Haliotis rubra*) and spiny lobsters (*Jasus edwardsii*) were monitored (Johnson et al. 2013; Ling & Keane^2021^), the relatively small and subordinate periwinkles were not routinely surveyed but were commonly encountered as scattered individuals residing on the benthos or on fronds of the kelp *Ecklonia radiata* (see also Shepherd & Edgar 2013). Notably, periwinkles have also been the target of commercial dive fisheries across south-east Australia since the 1980s, with a minimum legal-size limit of 45-mm shell width applied for the Tasmanian fishery (Keane et al. 2014). Indicating a broader presence of peri-barrens across eastern Tasmania, localised subtidal peri-barrens of 10 s of square metres in size were also observed at a site north of Bicheno in 2022 at a depth of 9 m (Fig. 1a, c) and in the shallow subtidal at a depth of 1–2 m at Pirates Bay in southeastern Tasmania (Fig. 1a; SD Ling, *pers. obs.*).

Size frequency and abundance of the *L. undulata* population on the ostensible peri-barren were surveyed on 16 July 2023, approximately one month prior to the commencement of the caging experiment. Size-graded counts within 10-mm size bins, from 25 to 75 mm, were conducted within twenty 0.5-m by 0.5-m quadrats systematically positioned across the peri-barren (e.g. Fig. 1d). A tape measure was reeled out along the full length of the peri-barren with the top left corner of quadrats placed every meter along the transect. Additionally, ten photo-quadrats approximately 70 × 100 cm in dimension were taken at 2-m intervals along the tape measure, from which cover of standing fleshy macroalgae was scored by overlaying a 100-point semi-transparent grid centred on each image.

### Caging experiment

**Fig. 2:**
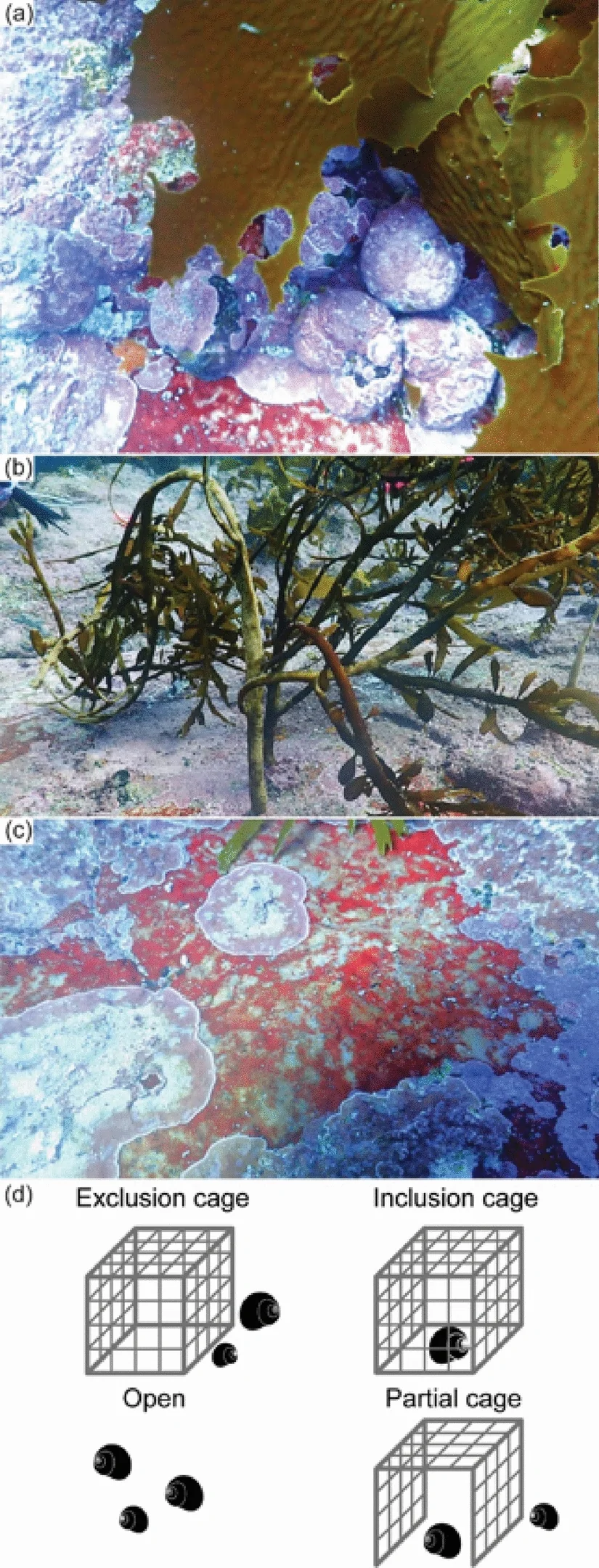
Apparent grazing impacts by hyperabundant periwinkles on standing fleshy and encrusting algae at 9-m depth, St. Helens Island 16 July 2023. **a** Ragged circular bite marks on *Ecklonia radiata* fronds with large periwinkles in foreground; note that fish bites from the locally present herbivorous herring cale (*Olisthops cyanomelas*) involve clean semi-circular or circular edges (SD Ling, *pers. obs.*), **b** pale scrape marks on stipe of *Phyllospora comosa*, **c** pockmarks on the bloodred encrusting *Peyssonnelia* sp. amongst pink crustose coralline algae, the latter appeared unimpacted (photographs SD Ling). **d** Schematic of the caging experimental design deployed on the local peri-barren comprising inclusion cages, exclusion cages, partial cages, and open plots (four replicates per treatment). Note the flatness of the rock in **c** which enabled cages to be squarely bolted to the reef to achieve a strong seal along the cage edges

Experiments utilising cages to contain and/or exclude herbivorous benthic invertebrates provide a powerful tool enabling the influence of a particular species on community dynamics to be rigorously determined (reviewed by Poore et al. 2012). To critically test the hypothesis that periwinkles can exert a top-down grazing influence on kelp-dominated reefs, we designed a caging experiment comprising *L. undulata* inclusion and exclusion treatments, along with open plots (background control), and partial cages (cage control), with 4 replicate plots per treatment equating to 16 experimental plots in total (Fig. 2d). The relatively low sample size was a product of logistical constraints experienced while undertaking a manipulative subtidal experiment in a remote, swell-prone location. Cages were constructed from plastic-coated galvanised wire which had been welded to form a 25-mm-square mesh. Cages were 125-mm cubes, containing a benthic reef area of 0.0156 m^2^. For inclusion cages, a single large periwinkle (~ 60 mm shell-width) was housed, equating to an effective density of 64 individuals m^−2^. This density was approximately 57% of the highest density for the resident hyperabundant population (i.e., 112 individuals m^−2^) and approximately 6-fold higher than the mean density (i.e., 11 individuals m^−2^) observed within systematic quadrats on the local peri-barren in July 2023. Therefore, the experimental density in cages is more representative of a grazing front rather than an average density. Furthermore, given the scale of flat rock sections on the peri-barren for which an appropriate seal could be maintained along the underside of cages, plus the need to minimise drag during periods of large ocean swell, the 125-mm-cubed cages provided an effective design.

Experimental plots were established by drilling 8-mm holes into the granitic rock and setting marine-grade masonry anchors (Dynabolts™) into the reef. A coupling nut was then used to secure a 150-mm length of 6-mm stainless-steel threaded rod to the reef. Each threaded rod was assigned a position from 1 to 16, with caging experiment treatments randomly assigned to each position. For plots assigned to full cages (periwinkle inclusion and exclusion) and partial cages, cages were bolted down by placing the cage centrally over the threaded rod and tightening a flange nut over a numbered 3 × 3 cm plastic cap affixed to the top of the mesh using stainless screws. For threaded rods marking open plots, only a plastic numbered cap was affixed to the top of the thread.

The caging experiment was established on the peri-barren at St. Helens Island (Fig. 1a), on 21 August 2023. Experimental plots were initially checked after 13 days to confirm experimental treatments were being maintained as planned. Experimental assessments then occurred at 34, 88, 117 days after commencement of the treatments. The experiment naturally concluded on day 117 as an assessment planned on day 174 was abandoned as the cage treatments had become compromised following invasion by juvenile periwinkles (shell width less than the 25-mm cage mesh), which had recruited into the cages.

Assessment of experimental plots involved unscrewing caps and cages from the central threaded rod and taking a photograph squarely above the benthos centred on the threaded rod. Percentage cover of fleshy macroalgae (inclusive of all foliose red, green and brown algae), filamentous turfing algae (hereafter ‘turf’), crustose coralline algae (CCA), and bare rock was scored using image analysis of the photograph by overlaying a 100-point semi-transparent grid over the 125 × 125 mm plot boundaries centred over each threaded rod. During each assessment period, cages were cleaned of fouling using nylon scrubbing brushes. For the inclusion caging treatment, individual periwinkles were replaced by new individuals during each assessment to reduce risk of potential caging-induced death from starvation, which would have resulted in complete loss of the grazing treatment. That is, three different individuals occurred within each inclusion cage during the experiment with an average of 39-days containment for each individual, with a total of 12 individuals contained for the cage inclusion treatment over the 117-day experiment.

### Repeat survey of periwinkle size, abundance and local algal cover

During the 117-day caging experiment, larger size classes of periwinkles (> 55 mm shell width) disappeared from the peri-barren consistent with systematic commercial harvesting of legal-sized individuals (> 45 mm shell width). Corroborating this local reduction of large individuals at our study site, commercial catch of periwinkles for the St. Helens Island reporting sub-block was ~2.4 tonnes during the 2022/23 fishing season (J. Keane 2025, pers. comm.). Given this dramatic local decline in legal-sized periwinkles, a repeat survey of periwinkle size and abundance as well as cover of standing fleshy macroalgae on the same reef area using the same methods and number of quadrats was then conducted on 26 November 2024, 499 days (~ 16 months) after the initial baseline sampling event. Wet-weight biomass estimates for *L. undulata* were established for both baseline and repeat surveys using the allometric conversion: *Wt* = 0.000275 × *L*^3.018 (*Wt* = wet weight in grams; *L*= length in mm, after Keane et al. 2014).

### Data analysis

The cover of fleshy algae, turf, crustose coralline algae (CCA), and bare rock were compared between treatments at the conclusion of the experiment (i.e., at Day 117) using separate generalised linear models (GLMs). In all cases, the cover of the specific benthic category was treated as the response variable, while treatment (four levels) was fitted as a fixed categorical effect. The models were based on a beta-binomial distribution with a logit-link function, which was well-suited to such proportional cover data (Harrison 2015). However, the model for bare-rock cover failed to converge with the beta-binomial distribution as some factor levels were based entirely on zero observations. In this case a beta distribution with a logit-link function was used with the addition of a small constant (0.001) to account for the zeroes present in the dataset. Model fit and assumptions were evaluated based on simulation-based model checking procedures [i.e., QQ plot, residuals versus predicted plot, and dispersion test; package *DHARMa* (Hartig 2020)]. Post-hoc pairwise comparisons of means, based on a Tukey’s correction, were also conducted to examine differences between treatment levels.

Variation in the abundance and biomass of *L. undulata* as surveyed in twenty 0.25 m^2^ quadrats on the peri-barrens during two periods, i.e. prior and post-caging experiment, were examined using separate GLMs. In these GLMs, the abundance (both above and below the legal fishery size of 45-mm shell width) and biomass of *L. undulata* were treated as response variables, while sampling time (two levels) was treated as a categorical fixed effect. The abundance models were based on a negative binomial distribution with a log-link function, while the biomass model was based on a Tweedie distribution with a log-link function. Variation in the cover of fleshy algae over time was examined using a GLM based on a beta-binomial distribution with a logit-link function. In this case, sampling time (two levels) was again treated as a categorical fixed effect. Model fit and assumptions were evaluated using simulation-based model checking procedures, as above. All statistical analyses were performed in *R* (R Core Team 2024; version 4.2.2), using the *tidyverse* (Wickham et al. 2019), *glmmTMB* (Brooks et al. 2017), and *emmeans* (Lenth 2020) packages.

## Results

### Caging experiment

**Fig. 3:**
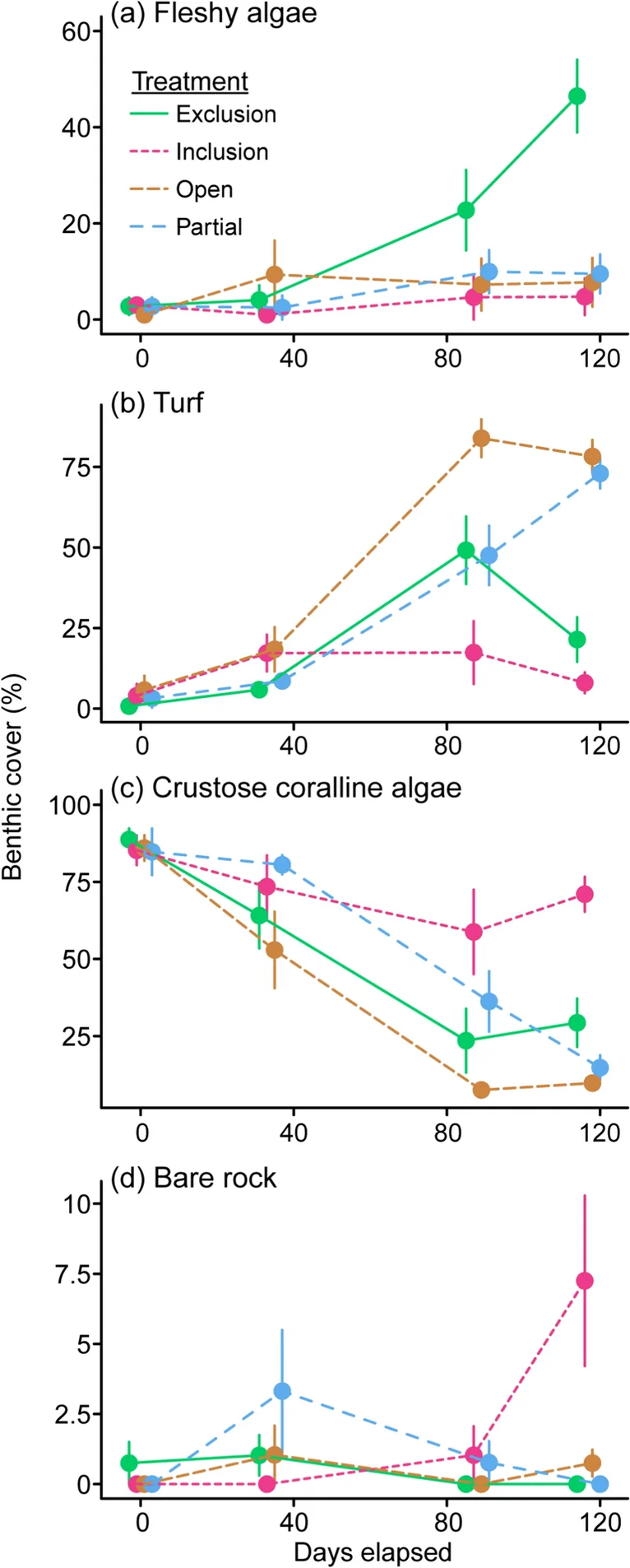
Temporal trends in benthic cover of **a** fleshy algae, **b** turfs, **c** crustose coralline algae, and **d** bare rock exposed to grazing by *Lunella undulata* under different caging treatments. In all cases the point and ranges denote the mean ± standard error from the raw data (four replicates per treatment per time period, N = 64) jittered at the start and at 34, 88, 117 days after commencement of the treatments

There were distinct temporal trends in the cover of different algae and bare rock in each of the four experimental treatments, with a clear influence of *L. undulata* on the reef community at the natural cessation of the experiment after 117 days (Fig. 3). In terms of fleshy algae, this benthic component was predominantly composed of fleshy brown understorey algae (Fig. S2), which only proliferated substantially in the exclusion cages where there was no grazing by *L. undulata* (Fig. 3a). After 117 days, the GLM revealed that mean (± SE) fleshy-algae cover was 47 ± 8% in exclusion cages, which was 5-fold, 6-fold, and 10-fold higher than in partially caged plots, open plots, and inclusion cages, respectively. Based on the GLM, the cover of fleshy algae was significantly higher (*P* < 0.01 in all three cases; Table S2) in the exclusion treatment compared to all other treatments at Day 117.

The cover of turfs varied between treatments at the 117-day mark depending on the intensity of grazing by *L. undulata* (Fig. 3b). Open and partially caged plots experienced intermediate levels of grazing and supported the highest turf cover after 117 days (≥ 73% on average in both cases), with this cover being significantly higher than in inclusion and exclusion cages (GLM, *P* < 0.0001 in all cases; Table S2). In the intensively grazed inclusion-cage treatment, turf cover did not proliferate substantially remaining at less than 18%, on average, at all sampling times (Fig. 3b). In exclusion cages with no grazing by *L. undulata*, turf initially proliferated and peaked at 49 ± 11% cover after 88-days. However, by Day 117, turf cover had declined to 22 ± 7% in exclusion cages, and by this time turf cover was not different to that in inclusion cages (GLM, *P* = 0.158; Table S2).

While there was no difference in turf cover between exclusion and inclusion cages by the end of the experiment, which represent the two extremes of grazing intensity, the differences between these treatments manifested in terms of the cover of crustose coralline algae (CCA) and bare rock (Fig. 3c, d). Specifically, initial proliferation of turf cover in exclusion cages gave way to proliferation of fleshy algae in the absence of grazing in this treatment (Fig. 2a, b). In contrast, turf cover never proliferated in the heavily grazed inclusion cages, with CCA cover dominating in this treatment (Fig. 2b, c). After 117 days, CCA cover had remained high (71 ± 6%) in the inclusion cages, with this cover being higher (by 2–7-fold) than in all other treatments (GLM, *P* <0.0001 in all cases; Table S2). In the other three treatments, CCA was largely overgrown by turfing and/or fleshy algae by Day 117 (Fig. 3c). Similarly, the only treatment where the cover of bare rock had increased markedly was in the heavily grazed inclusion cages (Fig. 3d), and after 117 days bare rock cover in inclusion cages was significantly higher than in all other treatments (GLM, *P* <0.01 in all three cases; Table S2).

### Broader ecosystem changes

**Fig. 4:**
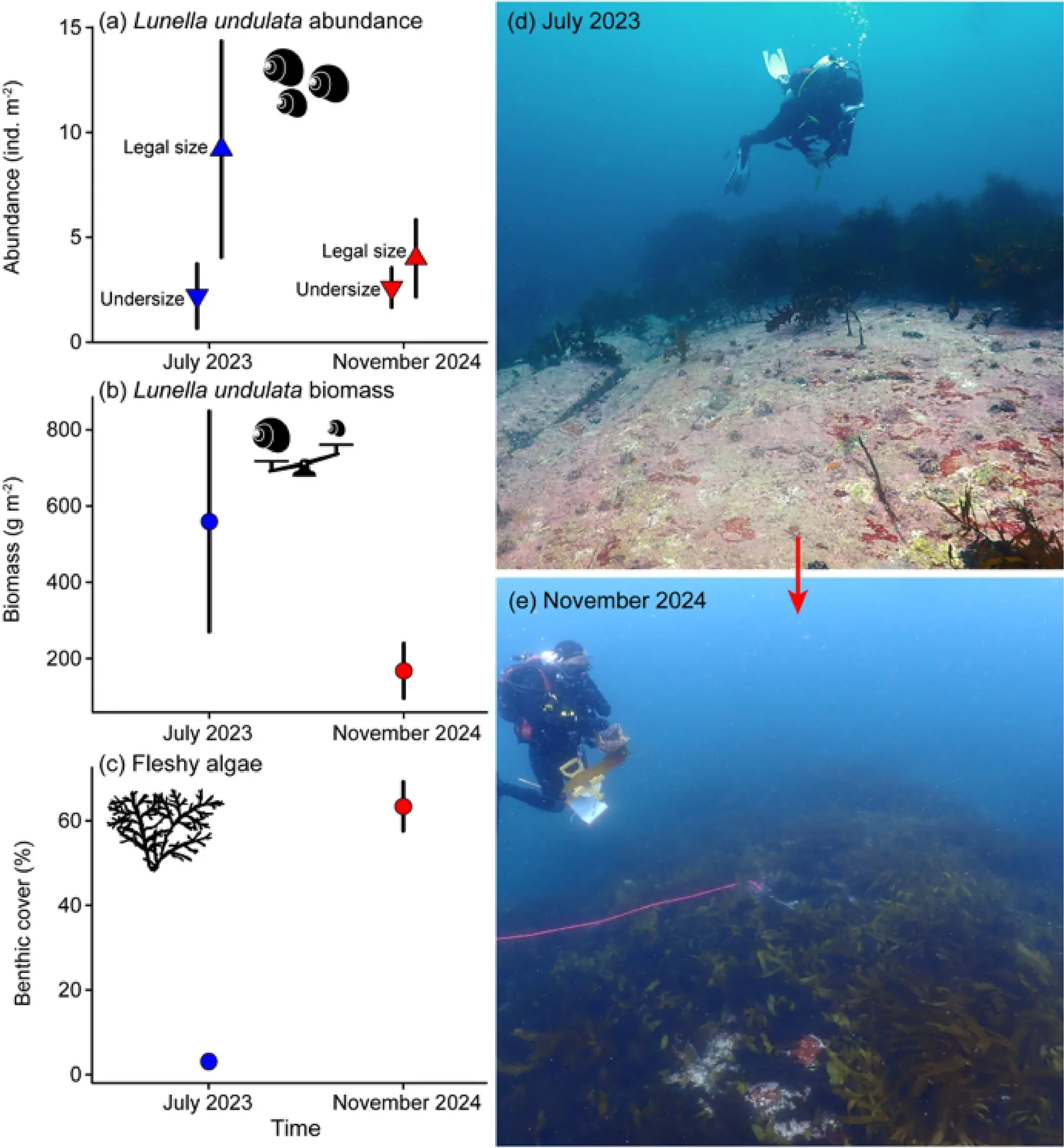
Variation in the **a** abundance of *Lunella undulata* above and below the minimum fishery size limit of 45-mm maximum shell width, **b** biomass of *L. undulata*, and **c** fleshy algae cover, through time in an *L. undulata* barren at St. Helens Island, Tasmania. In all cases the point and ranges denote the mean ± standard error from the raw quadrat data (twenty replicates per time period, N = 40). Photographs showing **d** the low cover of fleshy algae in the *L. undulata* ‘peri-barren’ on 15th July 2023, and **e** high fleshy algae cover in the same location on 26th November 2024 after decrease in *L. undulata* biomass (photographs SD Ling)

The original overgrazed *L. undulata* barrens at St. Helens Island where the caging experiment was conducted underwent marked ecological changes over the ~16-month period between July 2023 and November 2024 (Fig. 4). The overall mean (± SE) density of *L. undulata* fell from 11 ± 6 individuals m^−2^ to 7 ± 2 individuals m^−2^ over this ~16-month period. While there was no significant change in the mean density of undersize or legal-size *L. undulata* over this time (Table S3), larger periwinkles (> 55 mm shell-length) were absent at final sampling with all legal-size periwinkles measuring between 45 and 55 mm, consistent with apparent local harvest of larger legal-sized individuals. In contrast, initially more than half (52%) of all legal-sized individuals were > 55 mm, while 15% of initial legal-sized individuals were > 65 mm in length. Overall, there was a 3-fold decrease in the average biomass of *L. undulata* between sampling periods (from 559 g m^−2^ ± 289 SE to 168 g m^−2^ ± 71 SE, although this was not significant; GLM, *P* = 0.106; Fig. 4b; Table S3), equating to an estimated absolute decline of ~ 19 kg of benthic herbivore biomass across the ~48 m^2^ peri-barren. Over the same time, the cover of fleshy algae in the barrens increased by ~ 20-fold from 3 ± 0.02% to 63 ± 6% (GLM, *P* < 0.0001, Fig. 4c; Table S3).

## Discussion

### Confirming causation of peri-barrens

Inclusive of field observations, surveys and a critical manipulative experiment, our results demonstrate the capacity of the periwinkle *Lunella undulata* to impact subtidal reef community dynamics when occurring in localised hyperabundance at our study site. Building on previous inconclusive experimental results (Fletcher 1987), our results provide causation for prior correlations between bare patches of subtidal reef and hyperabundant periwinkle populations (Keough et al. 1993; Vanderklift and Kendrick 2004; Currie and Sorokin 2009; Shepherd and Edgar 2013; Keane et al. 2014). Our results are also consistent with that of experimental manipulations of ‘periwinkles’ (*Littorina littorea*) in the intertidal zone of the northern hemisphere, whereby this grazing gastropod has been shown to prevent establishment of the leathery macroalga *Fucus* when occurring at unusually high abundances (Lubchenco 1983). Despite *Lunella* and *Littorina* sharing the common name ‘periwinkle’, they are not closely related gastropods (Order Trochida, Family Turbinidae versus Order Littorinimorpha, Family Littorinidae). This raises the potential for grazing impacts across a broader range of herbivorous gastropods, such as abalone (e.g., Valentine et al. 2010) and subtidal limpets (e.g., Fletcher 1987), when reaching local hyperabundance.

In our case, patterns of grazing by a typically subordinate gastropod, when occurring at hyperabundance, were broadly consistent with overgrazing caused by dominant urchin grazers (e.g., Filbee-Dexter and Scheibling 2014; Ling et al. 2015, 2018). Albeit crustose coralline algae appeared more intact and rugose in the presence of hyperabundant periwinkles (see Fig. 2a–c), as opposed to the obvious bite marks and flattened/etched appearance of CCA associated with intensively scrapping urchins (Steneck 1983). As noted by Lubchenco (1983), the highly localised grazing effect of hyperabundant periwinkles can be as obvious and dramatic as urchins or some tropical fishes (reviewed by Lubchenco and Gaines 1981; Gaines and Lubchenco 1982). This highlights the potential for subordinate grazers to dominate the critical biological process of herbivory under certain contexts.

### Peri-barren disappearance

Beyond the confines of the caging experiment, broader observations on the peri-barren over the 16-month study revealed dramatic community change as a result of the ‘natural’ experiment whereby apparent harvesting removed hyperabundance of large legal-sized periwinkles. From start to end of the study, periwinkle biomass declined 3-fold on the peri-barren (from ~ 560 to 170 g m^−2^), while fleshy macroalgae ultimately proliferated from near zero to a full recovery of kelp beds over ~ 500-days from July 2023 to November 2024. Unlike the destructive mode of grazing observed during hyperabundance (Fig. 2a–c), following biomass reduction direct consumption of standing kelp by periwinkles was not observed. Given the distinct shift in size-structure observed, it remains an open question as to whether larger adults are more capable of destructively consuming standing algae, or whether destructive grazing was purely a result of low algal food sources relative to herbivore biomass at the edge of the peri-barren. Notably, the same destructive grazing habit by large adults was also observed on an adjacent peri-barren atop a flat rock surface along the same depth contour approximately 40 m to the south, which also appeared to be simultaneously harvested and was similarly observed to fully recover to a kelp bed within the ~ 16 months (SD Ling, *pers. obs.*).

### Peri-barren maintenance versus formation

Here our experiment was designed to test the influence of periwinkles on algal communities and was established on already ostensibly overgrazed ‘peri-barren’ reef as opposed to being an explicit test of the density of periwinkles required to overgraze kelp beds and form peri-barrens in the first instance. That is, we did not enclose standing kelp within our experimental cages at differing initial densities or biomass of periwinkles, which indeed constitutes a follow-up experiment (see Kriegisch et al. 2016). Nevertheless, the visible evidence of destructive bite marks on standing macroalgae associated with periwinkle hyperabundance prior to apparent harvest (see Fig. 2a–c), indicates that a destructive mode of periwinkle grazing can occur at hyperabundance when a mean biomass of ~ 600 g m^−2^ is sustained. Interestingly, such a grazer biomass approximates the ~ 700 g urchins m^−2^ that is globally indicated to result in kelp bed overgrazing (Ling et al. 2015).

Representing the active grazing edge of the peri-barren, a maximum periwinkle biomass of ~ 6700 g m^−2^ was observed within a single quadrat which was more than twice macroalgal biomass estimated at ~ 2600 g m^−2^ based on a density of two *P. comosa* thalli m^−2^ (local mature *P. comosa* thalli have a mean wet weight of ~ 1300 g, SD Ling *unpub. data*). Such a skewed grazer to algal biomass ratio is typical of destructive urchin feeding fronts where urchins aggregate at the edge of barrens and progressively overgraze kelp beds (Dean et al. 1984; Scheibling et al. 1999; Gagnon et al. 2004; Lauzon-Guay and Scheibling 2007; Reeves et al. 2022). Thus, the hyperabundant aggregations of periwinkles at ~ 6,700 g m^−2^ that were observed to be directly consuming standing kelps at the edge of the peri-barren, suggests that these overgrazed patches may also advance by a similar mobile feeding-front mechanism. Furthermore, similar to observations of rapid advancement of urchin barrens coincident with dieback of large macroalgae at the edge of barrens patches, such dieback and the creation of temporary bare patches or ‘gaps’ within kelp beds that are free of intense macroalgal whiplash, may also facilitate the establishment and/ or expansion of peri-barrens (e.g., Konar and Estes 2003; Ling 2008).

### Patterns and drivers of hyperabundant aggregations

As previously reported, bare reef associated with hyperabundant periwinkles in our study area occurred at scales of 10 s to 100 s m^2^ (Keough et al. 1993; Shepherd and Edgar 2013; Keane et al. 2014) and typically occurred on flat rock reef surfaces [as also described intertidally in the northern hemisphere (Lubchenco 1983)]. Initially we postulated that the hyperabundance of large periwinkle adults may have been spawning aggregations. However, peak spawning activity in eastern Tasmania is during January and February (Keane et al. 2014), whereas the St. Helens Island aggregations were first noticed in March 2023 and were present through winter until apparent harvest of adult biomass, suggesting aggregations persist independently of spawning. Facilitating persistent aggregations, we also observed recruitment of juveniles during our experiment which were likely to have come from adults around the isolated St. Helens Island given the short non-feeding larval stage of *L. undulata* that drift in the water column for only a few days before settlement (Shepherd and Edgar 2013). Thus, high local recruitment success and strong post-settlement survival through to large adult sizes, plus localised aggregation, likely enables hyperabundance to arise but which would require multiple years to build-up based on our observations and reported growth rates of ~ 2.8 to 3.7 years to reach the 45 mm shell width legal-size (Keane et al. 2014).

### Reduced top-down forcing and competitive release of a subordinate grazer?

The increasing dominance of the range-extending *C. rodgersii* across eastern Tasmania has been recognised as a significant threat to *L. undulata* populations and the commercial periwinkle fishery itself (Keane et al. 2014). Raising this concern, manipulative field experiments by Strain and Johnson demonstrated that *C. rodgersii* is a competitive dominant over other herbivores including the abalone *Haliotis rubra* and the native short-spined urchin *Heliocidaris erythrogramma*, with both these subordinate herbivores experiencing reduced algal food sources and cryptic behaviour in the presence of this dominant urchin (Strain and Johnson 2009, 2013; see also Andrew 1989). Given increased harvest of *C. rodgersii* in the St. Helens region in recent years (Cresswell et al. 2025), abundance of this urchin has been driven down on shallower intensively fished reefs < 12 m depth, where kelp recovery has occurred along the shallowest margins of kelp beds at the St. Helens Is. study site (SD Ling, *unpub. data*). Despite reduced urchin abundances along the shallow edges of contemporary urchin barrens, urchin biomass still largely occurs above the threshold for kelp recovery (~ 70 g urchins m^−2^; Ling et al. 2015) and here the cover of turf algae has proliferated (Ruiz-Ruiz et al. 2025). It is along this now turf-dominated edge that *L. undulata* recruitment appears high (Fig. S3), suggesting that the dominant urchin grazer may have contributed to the local population irruption of the subordinate periwinkle by opening reef surfaces to sunlight for rapid microalgal growth, which can be grazed efficiently by gastropods (e.g., Fletcher 1987). Thus, we hypothesise that competitive release from *C. rodgersii,* due to increased local harvesting of this urchin, has facilitated greater access to turf algal resources at this site, allowing the periwinkle to reach local hyperabundance and effect overgrazing within adjacent kelp beds.

The emergence of peri-barrens at the study site has occurred in the functional absence of natural periwinkle predators, chiefly the spiny lobster (*Jasus edwardsii*). Lobsters consume *C. rodgersii* and *H. erythrogramma* urchins (Pederson & Johnson 2006; Ling et al. 2009) plus the periwinkle *L. undulata* which was consumed at a high rate on urchin barrens nearby St. Helens Island (Smith et al. 2023). Thus, predatory release of both urchins and gastropods will favour herbivore hyperabundance and increased herbivory (Pederson and Johnson 2006; Ling et al. 2009; Ling and Johnson 2012). While predatory lobsters target smaller size-classes of herbivore prey (Ling et al. 2009; Ling and Johnson 2012), harvesting of herbivores targets larger legal-size individuals and harvest-driven declines of periwinkles in eastern Tasmania appears stronger than natural predation based on long-term declines of periwinkles on fished reefs relative to increases in periwinkles inside Marine Protected Areas over a 30-year period (Ling et al. 2021).

Considering perspectives from deeper time, hyperabundant intertidal/ shallow subtidal aggregations of periwinkles were very likely to have occurred over millennia on northeastern Tasmanian reefs given the dominance of *L. undulata* (known as Warrener Shell) in Aboriginal middens standing several metres high and carbon dated to ~ 6 K years BP (Lourandos 1968; see also Sherwood 2019). Likely associated with hyperabundant intertidal/ shallow-water harvestable populations, we infer that localised peri-barrens were also a common feature during the era when shell middens accumulated. Notably, middens elsewhere in Tasmania indicate initial consumption of fishes and crustaceans (lobsters) in deeper layers, followed by dominance of mollusc shells (reviewed by Jones 1995). Such local sequential harvest of large higher trophic-level predatory spiny lobsters (Ling et al. 2009), to abalone (McAllister and Mundy 2024), to urchins (Cresswell et al. 2025), and then periwinkles as the smallest of all commercially harvested reef species, is now apparent for northeastern Tasmanian reefs which is consistent with the notion of fishing-down food webs reported globally (Pauly et al. 1998; see also Jackson et al. 2001).

## Conclusions

In the absence of hyperabundant urchin populations over hundreds of hectares, here we have revealed a highly localised influence of subordinate herbivores that can also be inferred from landscape patterns. Unlike highly mobile herds, localised spatially constrained intense herbivory by benthic invertebrates can maintain grazed areas leading to persistent alternative stable states of reef communities (Ling et al. 2015). In the case of peri-barrens, the emergence of this phenomenon at our long-term experimental study site was seemingly a fleeting moment during broader multi-decadal natural history observations. With a highly localised rise to dominance and impact on the reef community, followed by systematic harvesting and then algal recovery, the phenomenon was gone too soon to determine whether the periwinkles had ‘eaten themselves out of house and home’, or whether the emergent peri-barrens were to ultimately demonstrate stability as an alternative reef state. While the dominant urchin grazers are undoubtedly structuring the reef over great extent, the local rise of a subordinate herbivore to dominance is of ecological interest. Such unanticipated ‘ecological surprises’ are potentially symptomatic of ongoing cascading changes driven by climate change coupled with ongoing intensive harvesting of a multitude of reef species which are being sequentially depleted from reefs at local to global scales.

## Supplementary Information

Below is the link to the electronic supplementary material.

Supplementary file1 (DOCX 11023 KB)

Supplementary file2 (XLSX 22 KB)

Supplementary file3 (RMD 11 KB)

## Data Availability

All raw data are provided as Electronic Supplementary Material.

## Code availability

All code is provided as Electronic Supplementary Material.
