# Supplementary material for: Hyperabundance of a subordinate herbivorous gastropod causes localised overgrazing within kelp beds
Source: Oecologia. 2026 Sep 6;208(10):131. doi: 10.1007/s00442-026-05970-x (PMC13547173; doi:10.1007/s00442-026-05970-x)
Supplement: Supplementary file 1 [file 442_2026_5970_MOESM1_ESM.docx]

**Electronic supplementary material**

**Hyperabundance of a subordinate herbivorous gastropod causes localised overgrazing within kelp beds**

Scott D. Ling^*^, Matthew Rose, Paula Ruiz-Ruiz, Sterling B. Tebbett

^1^Institute for Marine and Antarctic Studies, University of Tasmania, Hobart, Tasmania, Australia 7004

*Scott.Ling@utas.edu.au

**Table S1** Summary of results from generalised linear models (GLMs) used to examine the effects of caging treatments on different benthic covers at the end of the experiment (day 117). SE = standard error, CCA = crustose coralline algae.

| **Response variable** | **Model used** | **Predictor variable** | **Estimate** | **SE** | ***z* value** | ***p* value** |
| --- | --- | --- | --- | --- | --- | --- |
|  |  |  |  |  |  |  |
| **Fleshy algae cover** | Beta-binomial GLM | Intercept  Inclusion  Open  Partial | -0.142  -3.180  -2.578  -2.000 | 0.312  0.774  0.648  0.557 | -0.456  -4.108  -3.977  -3.595 | 0.649  <0.0001  <0.0001  <0.001 |
|  |  |  |  |  |  |  |
| **Turf cover** | Beta-binomial GLM | Intercept  Inclusion  Open  Partial | -1.322  -1.096  2.603  2.296 | 0.265  0.457  0.374  0.361 | -4.982  -2.397  6.959  6.367 | <0.0001  0.017  <0.0001  <0.0001 |
|  |  |  |  |  |  |  |
| **CCA cover** | Beta-binomial GLM | Intercept  Inclusion  Open  Partial | -0.901  1.804  -1.204  -0.841 | 0.236  0.333  0.403  0.375 | -3.824  5.415  -2.990  -2.242 | <0.001  <0.0001  0.003  0.025 |
|  |  |  |  |  |  |  |
| **Bare rock cover** | Beta GLM | Intercept  Inclusion  Open  Partial | -5.164  2.489  0.550  0.000 | 0.550  0.578  0.642  0.662 | -9.390  4.307  0.857  0.000 | <0.0001  <0.0001  0.391  1.000 |
|  |  |  |  |  |  |  |

**Table S2** Results of post-hoc means comparisons for generalised linear models (GLMs) used to examine the effects of caging treatments on different benthic covers at the end of the experiment (day 117). SE = standard error, CCA = crustose coralline algae.

| **Benthic cover** | **Comparison** | **Estimate** | **SE** | ***z* ratio** | ***p* value** |
| --- | --- | --- | --- | --- | --- |
|  |  |  |  |  |  |
| **Fleshy algae** | Exclusion – Inclusion  Exclusion – Open  Exclusion – Partial  Inclusion – Open  Inclusion – Partial  Open – Partial | 3.180  2.578  2.000  -0.602  -1.180  -0.577 | 0.774  0.648  0.556  0.880  0.817  0.704 | 4.108  3.977  3.595  -0.684  -1.444  -0.820 | <0.001  <0.001  0.002  0.903  0.472  0.845 |
|  |  |  |  |  |  |
| **Algal turf** | Exclusion – Inclusion  Exclusion – Open  Exclusion – Partial  Inclusion – Open  Inclusion – Partial  Open – Partial | 1.096  -2.603  -2.296  -3.699  -3.392  0.307 | 0.457  0.374  0.361  0.462  0.451  0.356 | 2.397  -6.959  -6.367  -8.004  -7.523  0.862 | 0.078  <0.0001  <0.0001  <0.0001  <0.0001  0.825 |
|  |  |  |  |  |  |
| **CCA** | Exclusion – Inclusion  Exclusion – Open  Exclusion – Partial  Inclusion – Open  Inclusion – Partial  Open – Partial | -1.804  1.204  0.841  3.008  2.644  -0.364 | 0.333  0.403  0.375  0.406  0.377  0.436 | -5.415  2.990  2.242  7.409  7.018  -0.835 | <0.0001  0.015  0.112  <0.0001  <0.0001  0.838 |
|  |  |  |  |  |  |
| **Bare rock** | Exclusion – Inclusion  Exclusion – Open  Exclusion – Partial  Inclusion – Open  Inclusion – Partial  Open – Partial | -2.490  -0.550  0.000  1.940  2.490  0.550 | 0.578  0.642  0.662  0.524  0.578  0.642 | -4.307  -0.857  0.000  3.703  4.307  0.857 | <0.0001  0.827  1.000  0.001  <0.0001  0.827 |

**Table S3** Summary of results from generalised linear models (GLMs) used to examine the abundance and biomass of *L. undulata* and the cover of fleshy algae on the reef. SE = standard error

| **Response variable** | **Model used** | **Predictor variable** | **Estimate** | **SE** | ***z* value** | ***p* value** |
| --- | --- | --- | --- | --- | --- | --- |
|  |  |  |  |  |  |  |
| **Undersize *L. undulata* abundance** | Negative binomial GLM | Intercept  November 2024 | 0.789  0.167 | 0.814  1.149 | 0.969  0.145 | 0.333  0.884 |
|  |  |  |  |  |  |  |
| **Legal size *L. undulata* abundance** | Negative binomial GLM | Intercept  November 2024 | 2.219  -0.833 | 0.753  1.069 | 2.946  -0.779 | 0.003  0.436 |
|  |  |  |  |  |  |  |
| ***L. undulata* biomass** | Negative binomial GLM | Intercept  November 2024 | 6.327  -1.202 | 0.459  0.744 | 13.786  -1.615 | <0.0001  0.106 |
|  |  |  |  |  |  |  |
| **Fleshy algae cover** | Beta-binomial GLM | Intercept  November 2024 | -3.212  3.801 | 0.502  0.573 | -6.404  6.632 | <0.0001  <0.0001 |
|  |  |  |  |  |  |  |

**Supplementary Fig S1** Map of St. Helens Island study site within southeastern Australia (a.); Google Earth view of St. Helens region with pin indicating precise latitude and longitude of the studied peri-barren (b.).

**Supplementary Fig S2** Proportion of fleshy algal types summed across four replicates within each treatment for each time-period (total N = 64) as indicated by the number of days post commencement of the caging experiment, commencing 21^st^ of August 2023. Fleshy ‘Brown’ understory algae (including *Dictyota, Dilophus, Dictyopteris, Halopteris, Colpomenia*, and *Zonaria*) dominated the fleshy algal cover through time. **‘**Red’ fleshy algae featured sporadically, while canopy-forming browns (*Ecklonia radiata* and *Phyllospora comosa*) contributed to the fleshy algal cover in the final sampling period only. The succession from more ephemeral fleshy browns towards canopy-formers by the end of the caging experiment was an indication of ultimate succession to kelp beds as the whole peri-barren recovered and was dominated by canopy-formers by end of the experiment.

**Supplementary Fig S3** High abundance of periwinkle recruits on high turf covered rock on the *C. rodgersii* barren at St. Helens Island, scalebar next to an individual *L. undulatus* is 20 mm in length. Note that this barren was scraped clean during high densities of *C. rodgersii* which have now been locally fished down but not to the point enabling kelp bed recovery (photo-credit SD Ling).
